# Age of tobacco, nicotine and cannabis use initiation in Switzerland: a sequence analysis among adolescents and young adults

**DOI:** 10.1186/s12889-024-20731-2

**Published:** 2024-11-19

**Authors:** Diana Fernandes, Lorraine Chok, Jérémy Cros, Luc Lebon, Karin Zurcher, Alexandre Dubuis, Cathy Berthouzoz, André Berchtold, Yara Barrense-Dias

**Affiliations:** 1 Department of Epidemiology and Health Systems, Research Group on Adolescent Health, Center for Primary Care and Public Health (Unisanté), University of Lausanne, Lausanne, Switzerland; 2 Department of Health Promotion and Prevention, Center for Primary Care and Public Health (Unisanté), University of Lausanne, Lausanne, Switzerland; 3 Promotion santé Valais, Sion, Switzerland; 4 Institute of Social Sciences & NCCR LIVES, University of Lausanne, Lausanne, Switzerland; 5 Département épidémiologie et systèmes de santé, Centre universitaire de médecine générale et santé publique (Unisanté), Groupe de Recherche sur la Santé des Adolescents (GRSA), Route de la Corniche 10, Lausanne, CH 1010 Switzerland

**Keywords:** Tobacco products, Cigarettes, Adolescents, Initiation age, Substance use

## Abstract

**Background:**

To explore at what age youth start using tobacco and/or nicotine products, which product is used first, product initiation sequences, and whether some socio-demographic characteristics are associated with substance use initiation.

**Methods:**

Data were collected from an online questionnaire disseminated through social media and professional partners, targeting youth aged 14–25 in French-speaking Switzerland in August 2022. The final sample included 1362 participants. Respondents were asked whether they had already used cigarettes, e-cigarettes, hookah, snus, or cannabis at least once in their life (yes/no) and the age of the first time was asked for each substance when the answer was positive. Additionally, participants were asked about their substance use in the past 30 days. Respondents were classified according to age at onset of each tobacco/nicotine and cannabis product, and comparisons were made based on sociodemographic variables, including gender (cisgender female/cisgender male/transgender), perceived socioeconomic status (below average/average/above average), and age.

**Results:**

Overall, in addition to those who reported no consumption, four distinct initiation profiles emerged from the classification. Cigarettes remain the most commonly used first product with an average age of 15.7 years. While cigarettes and hookah are present in all profiles, the order of first consumption varies from one profile to another, with cigarettes coming first in two profiles, hookah in one and e-cigarettes in the last. Furthermore, while the most common profile contains experimental consumption of the five products considered, some profiles do not contain cannabis, e-cigarettes and/or snus, for example. When divided by age groups, both 14-17-year-olds and 18-21-year-olds reported cigarette as their first product of initiation. Across the separate age groups (14–17, 18–21, 22–25), cigarettes consistently emerge as the primary used on average.

**Conclusions:**

Cigarettes remain the first product to be used, but the younger the respondents, the earlier they start using e-cigarettes, and very close to cigarettes. Regarding current consumption patterns, e-cigarettes are becoming increasingly prominent.

**Supplementary Information:**

The online version contains supplementary material available at 10.1186/s12889-024-20731-2.

## Introduction

Tobacco use is a major public health concern and the leading cause of premature death globally [1]. Smoking behavior generally begins during adolescence, often referred to as the experimentation period, and increases the risk of substance use disorders in adulthood [2, 3]. According to the World Health Organization, most smokers had already smoked a cigarette or were already addicted by the age of 18 [4]. A study based on data from 204 countries in 2019 found that 20.1% of males and 4.95% of females aged 15–24 were tobacco smokers (any type of smoked tobacco including manufactured cigarettes, cigars and shisha for example), with initiation ages ranging from 17 to 20 for current smokers in Europe [5]. In Switzerland, a study revealed that 28% of young people aged 15–24 were occasional or regular smokers of conventional cigarette in 2022. This prevalence is higher than in the whole population aged 15 and over (22%) [6].

In addition to cigarettes, multiple tobacco and/or nicotine products spark interest among young people such as electronic cigarettes (e-cigarettes) or electronic nicotine delivery systems, (ENDS) [7], hookah (or water pipe) and snus. The Health Behaviour in School-aged Children (HBSC) study [8] analysed substance use of 15-year-olds in the past 30 days. It was found that, from 2018 to 2022 e-cigarette use increased from 20.6 to 25.1% for boys, and from 12.9% up to 25% for girls. Snus use also increased and went from 6 to 12.8% for boys, and from 1.3 to 5.6% in girls [8]. Meanwhile, in the same period, conventional cigarette use was stable with 16% for girls (15.2–16.6%) and 15% for boys (16.6–14.4%) while use decreased by half for both girls (8–4.5%) and boys (14.2–8.9%). These trends were also reported between 2018 and 2022 in a study among Swiss men enrolled in army recruitment centers (mean age 20) [9]. Furthermore, a study conducted in two cantons in Switzerland among students aged between 15 and 21 years old in 2021–2022 showed that snus was the second product most likely to be used daily (4.1%) after cigarettes in commercial packages (14.2%) [10]. The arrival of disposable e-cigarettes around 2020, particularly popular among youth [11–13], may have contributed to the recent increase of e-cigarette use. In this line, the authors of a study on trends of adolescents’ tobacco use between 1999 and 2018 in 143 countries concluded that even with the decrease of the prevalence of cigarette smoking, the use of other tobacco products have increased or plateaued in a majority of countries [14].

Lastly, cannabis is another substance of interest for youths. In Switzerland, it can be legal (< 1% tetrahydrocannabinol, THC) or illegal (≥ 1% THC). As shown in the HBSC study, cannabis use among youth in Switzerland seems to be as stable as cigarettes with rates around 8% for girls and 13% for boys between 2018 and 2022. A stability in the age of initiation was also observed in France between 2000 and 2022, remaining just below 16 years [15, 16]. Precisely, some authors define the early age of cannabis initiation as 16-years or younger [17–19]. Continuous cannabis use has been attributed to early onset substance use, including tobacco products [2, 20]. On the contrary, odds of being a current cannabis smoker reduced with later onset cannabis smoking [20].

Access to a wide range of tobacco and/or nicotine products may encourage youths to engage in simultaneous use of multiple products [21, 22]. In Switzerland, 57% of youth aged between 14 and 25 years old reported using more than one tobacco and/or nicotine product in the last 30 days and 23% reported using both e-cigarettes and cigarettes in the last 30 days [12].

Few studies have explored the initiation age and the first tobacco and/or nicotine product used among youth. In Switzerland, it appears that cigarettes remain the primary entry point for tobacco consumption with 61% of young people aged 15 to 24 years old who smoke cigarettes started smoking with conventional cigarette. This rate is lower than the one for the overall population aged 15 and over (87%) [6]. Although the number of cigarette smokers reporting that they began with e-cigarettes remains low, this has only been observed among individuals aged 15 to 24, suggesting a possible generational change with younger users now having access to e-cigarettes.

A study conducted in 2018 in the city of Zurich on a cohort of young adults found that tobacco and cannabis had a median age-of-onset around 15 years [23]. In a study led in Norway [24], one of the countries with the highest prevalence of snus use among adolescents and young adults, the median age of snus initiation among 18-20-year-old participants was 16 years. To our knowledge, this is the first study on the initiation age of tobacco and/or nicotine products (including e-cigarettes and snus) in Switzerland. The aim of this study is to explore the age at which youth begin using tobacco and/or nicotine products, identify the first product used, examine product initiation sequences, and investigate whether some socio-demographic characteristics are associated with the initiation of tobacco and/or nicotine use. Such a study can contribute to reflections and understanding of the pattern of substance use during adolescence.

## Materials and methods

Data were obtained from an online questionnaire (10–15 min) conducted in the French-speaking part of Switzerland in August 2022 [12]. Participants were recruited by disseminating the survey on www.adosjobs.ch, a website dedicated to student jobs which is mainly accessed by high schoolers and youths having finished mandatory school, as well as through sponsored and non-sponsored ads on social media (Instagram, Facebook, and LinkedIn). Collecting data through social networks has known advantages already highlighted in literature; it reaches a bigger audience in a shorter period, especially younger populations [25–27]. The initial non-representativeness could be reduced through the calculation of weights taking into account three criteria whose exact distribution is known for the whole of French-speaking Switzerland through official census statistics: sex assigned at birth, age, and canton of residence. These weights were calculated on a final sample of 1362 participants. The sample used had no missing data.

### Measures

#### Initiation age

Respondents were asked whether they had already tried cigarettes, e-cigarettes, hookah, snus, or cannabis at least once in their life (yes/no) and in the past 30 days (yes/no). If they answered positively to lifetime use, they were asked at what age they had first tried that product. We included cannabis as tobacco is often added to the cannabis smoked as joints (“mulling”) [28, 29]. It is important to note that the question about conventional cigarettes also included the following tobacco products: cigars, cigarillos, and rolling tobacco. However, as only 3% of smokers aged 15–24 years-old have one of the last three products mentioned as their initiation product [6], they were grouped with cigarettes.

#### Sociodemographic characteristics

The chosen sociodemographic variables included gender (cisgender female/cisgender male/transgender), age at the time of the survey, and perceived socioeconomic status (below average/average/above average). That last variable was measured by asking participants how they perceived their family’ socioeconomic status compared to other families in Switzerland [30]. The sample was divided into three age groups (14–17, 18–21, and 22–25) considering the legal age in French-speaking Switzerland (18 years old) and the fact that the oldest respondents have had more years to experiment with different products and longer exposure to risk [31].

### Statistical analysis

All analyses were carried out globally, then separately for the three age ranges (14–17, 18–21 and 22–25). In the first stage, respondents who reported no use of nicotine products were set aside (*n* = 248) and the other data were classified into different groups according to age at first initiation of each nicotine product. Differences between individuals were calculated using a Euclidean distance and an agglomerative hierarchical classification was used [32]. The final groups were determined by the authors of the study based on theoretical and empirical considerations. Based on the dendrogram of agglomerative nesting, allowing a hierarchical clustering diagram that organizes data into a tree structure based on similarities, six groups emerged, but too small groups (containing less than ten people) were aggregated with other groups based on the similarity of sequences in order to keep sufficient statistical power for further analyses. Groups representing similar product initiation processes were aggregated as well. The result consists in four groups and this process was the same for the different age ranges, except for the 22–25 age group for which we retained five groups, each having enough participants. In the second stage, non-consumers were considered as an additional group, and the socio-demographic characteristics of each group, as well as the current consumption of individuals belonging to each group, were then determined. For the latter analyses, the data were weighted according to gender, age, and canton of residence to ensure that the sample structure would resemble that of young people in French-speaking Switzerland. However, differences remain due to a large disproportion in the number of respondents between certain groups in the target population. Every analysis conducted used the weighted sample to yield results that closely reflect reality. The only exception was the creation of initiation groups, as weights do not influence the hierarchical classification process used. Chi2 tests were carried out on weighted data for gender and socio-economic status, and ANOVA on weighted data for age, overall and for the three groups based on age. The significance level was set at 5%.

All statistical analyses were carried out using the free statistical software R (version 4.1.0) and the RStudio environment.

## Results

### Product and age of initiation

**Fig. 1:**
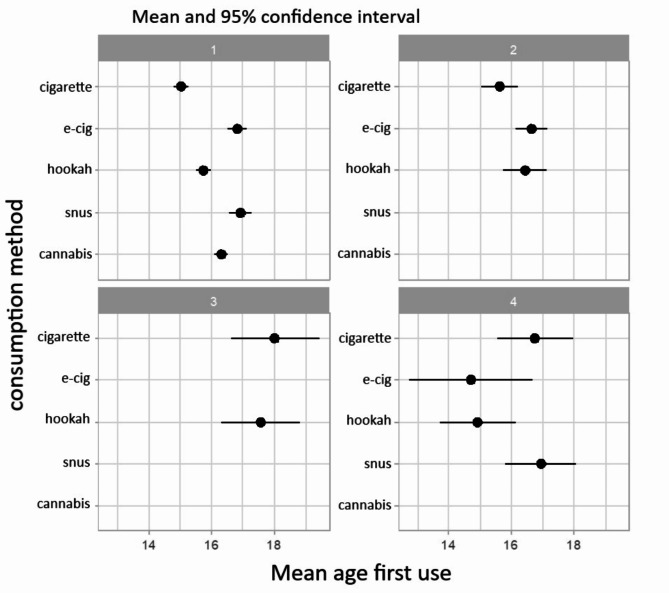
Mean age of each product used for the overall sample divided into four groups

The overall sample of 1362 people was divided into four groups (Table 1; Fig. 1), each representing a consumption profile differing in both product and age of initiation. It is important to note that not every person assigned to a group will have tried every substance mentioned. These groups represent typical behaviors with slight variations. Overall, mean age of initiation for all products was 15.7 years old and there were 248 “abstinent” youth that did not consume any of the tobacco/nicotine products. Group 1 (*n* = 688.2^1^) is the only group that has tried every substance at least once, and the first tobacco/nicotine product they used was cigarette (15 years), followed by hookah (15.7 years). The last product was snus (16.9 years). In Group 2 (*n* = 342.4), the first product ever tried was cigarette (15.6 years), followed by hookah (16.4 years) and e-cigarette (16.6 years) about a year later. Group 3 (*n* = 61.9) had hookah as the first product (17.6 years), very closely followed by cigarette (18 years). In Group 4 (*n* = 21.5), the first product tried was e-cigarette at a younger age (14.7 years) than in the other groups, followed closely by hookah. Cigarettes were tried later at 16.7 years, along with snus at 16.9 years.

**Table 1:** Demographic and substance use data (mean and %) by group for the overall sample

Overall		1	2	3	4	Abstinent	pvalue	
n		688.1796	342.4393	61.9078	21.4733	248.0000		
Age (mean)		20.4576	18.9200	21.8211	20.4726	18.2453	0.370	
Gender	Cisgender female	51.2	50.7	57.5	55.8	47.7	< 0.01	
	Cisgender male	43.3	45.4	42.5	44.2	48.8		
	Transgender	5.5	3.9	0	0	3.5		
Perceived socioeconomic status	Above average	21.7	15.3	24.8	33.1	18.1	< 0.05	
	Average	62.8	73.2	63.6	58.2	66.1		
	Below average	15.5	11.5	11.5	8.7	15.8		
Lifetime consumption	Cigarette	93.9	62.5	66.5	49.3	N/A		
	E-cigarette	89.7	100	0	20.7	N/A		
	Hookah	73.6	42.7	61.7	18.1	N/A		
	Snus	51.4	0	0	100	N/A		
	Cannabis	91.0	0	0	0	N/A		
30-days consumption	Cigarette	56.6	23.8	2.7	13.5	N/A		
	E-cigarette	56.0	54.1	0	9.6	N/A		
	Hookah	9.4	10.9	2.7	2.4	N/A		
	Snus	8.3	0	0	13.4	N/A		
	Cannabis	33.6	0	0	0	N/A		
	Ecig-cig dual	38.6	15.9	0	0	N/A		

*The groups refer to the groups from* Fig. 1

*Group 1 has tried all tobacco/nicotine products at least once – cigarette as the first product.*

*Group 2 has tried cigarette*,* e-cigarette*,* and hookah – cigarette as the first product.*

*Group 3 has tried cigarette and hookah with hookah as the first product.*

*Group 4 has tried all tobacco /nicotine products except cannabis with e-cigarette as the first product*

**Fig. 2:**
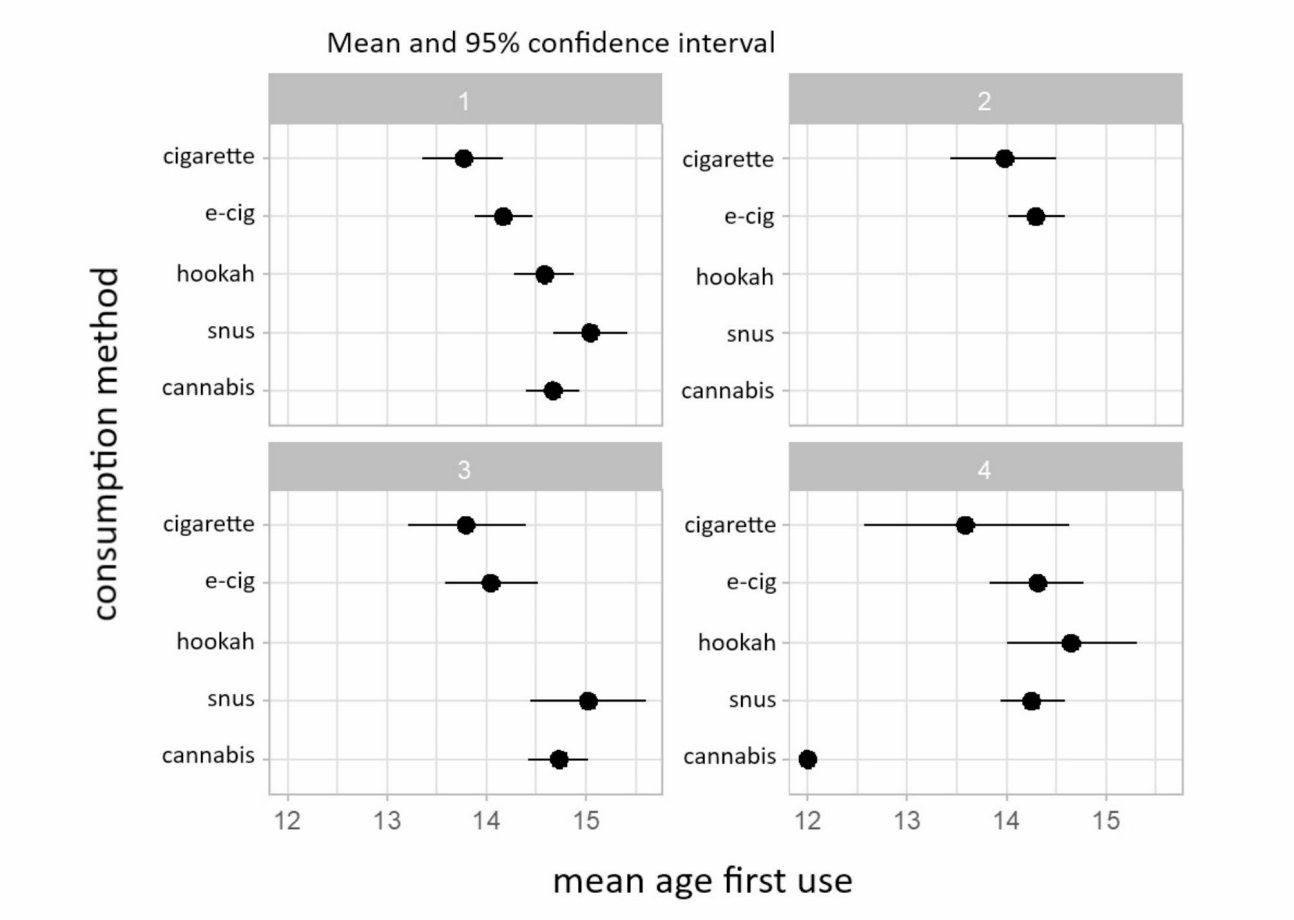
Mean age of each product used for the 14-17-year-olds divided into four groups

When looked at by age range, the results vary slightly. In the 14–17 age range (Table 2; Fig. 2), most groups had tried most, if not all, tobacco products by the age of 17. In Group 2 (*n* = 105.7), the largest group, only two substances have been tried: cigarette and e-cigarette. The cigarette was most commonly the first product tried by youth in each group (mean age 13.8), closely followed by the e-cigarette at around 14 years old. Group 4 (*n* = 29.1) is the exception, with cannabis being the first product ever tried at age 12; however this early cannabis use was reported by only one participant, suggesting exceptional behavior or trend.

**Table 2:** Demographic and substance use data (mean and %) by group for the 14-17-year-olds

		1	2	3	4	Abstinent	pvalue	
n		83.5286	105.6994	59.6825	29.0895	91.0000		
Age (mean)		16.2	15.3	15.7	15.5	15.2	0.07	
Gender	Cisgender female	40.6	52.4	48	62.2	43.5	< 0.01	
	Cisgender male	53.1	44.7	40.6	37.8	53.7		
	Transgender	6.3	2.9	11.4	0	2.8		
Perceived socioeconomic status	Above average	33	17.1	22	5.8	21.2	0.063	
	Average	53.9	73.5	66.3	81.5	71.2		
	Below average	13	9.4	11.7	12.7	7.6		
Lifetime consumption	Cigarette	96.8	37.23	86.3	50.8	N/A		
	E-cigarette	97.9	100	100	76.7	N/A		
	Hookah	100	0	0	40.2	N/A		
	Snus	48.9	0	33.4	54.3	N/A		
	Cannabis	69.2	0	100	1.6	N/A		
30-days consumption	Cigarette	66.6	11.8	60.5	33.4	N/A		
	E-cigarette	64.2	46.7	73.2	50.3	N/A		
	Hookah	22.7	0	0	5.6	N/A		
	Snus	6.6	0	4.4	13.3	N/A		
	Cannabis	43	0	46	1.6	N/A		
	Ecig-cig dual	50.4	9.5	48.9	21	N/A		

*The groups refer to the groups from* Fig. 2

*Group 1 has tried all nicotine / tobacco products with cigarette as the first product.*

*Group 2 has tried cigarette and e-cigarette with cigarette as the first product.*

*Group 3 has tried all nicotine / tobacco products except hookah with cigarette as the first product.*

*Group 4 has tried all nicotine / tobacco products with cannabis as the first product.*

**Fig. 3:**
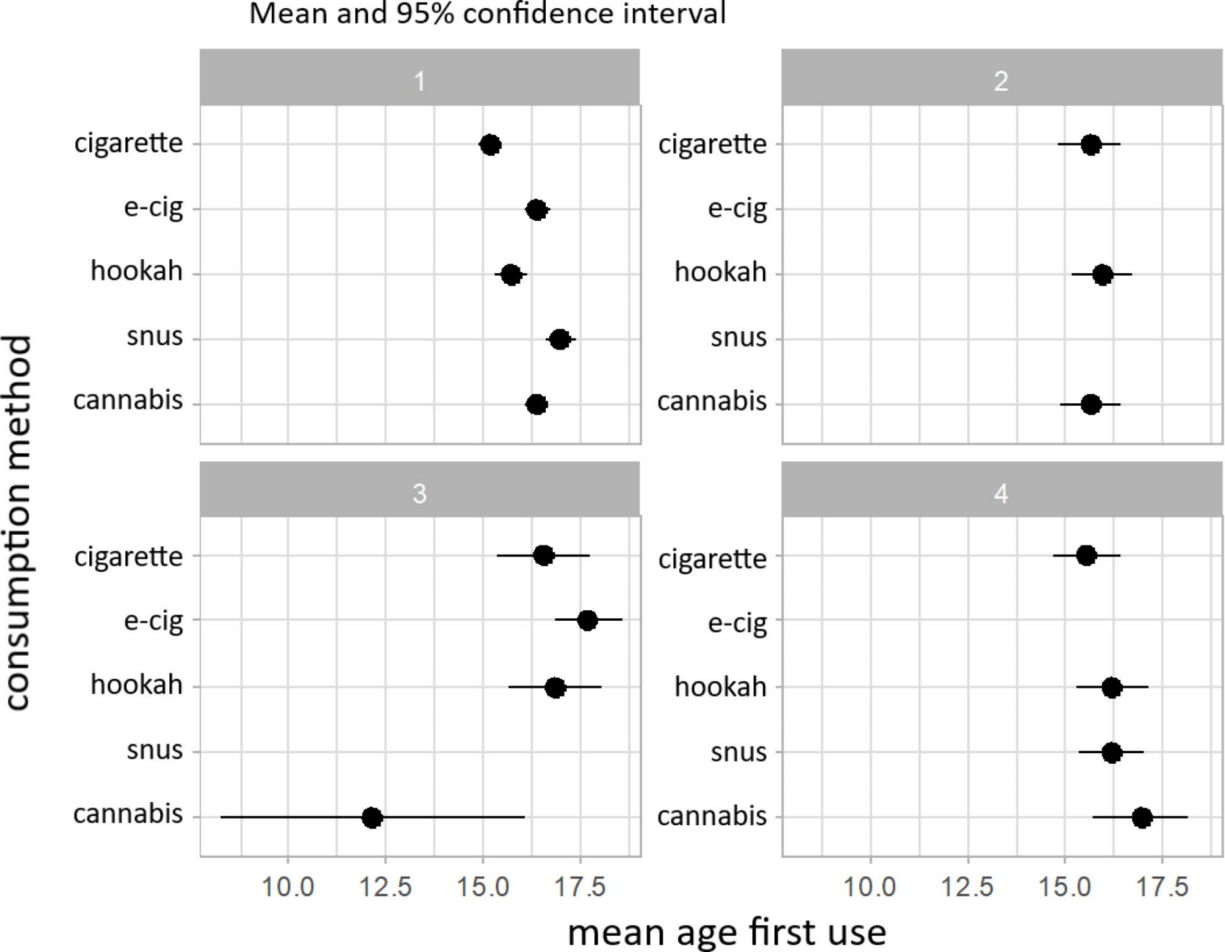
Mean age of each product used for the 18-21-year-olds divided into four groups

The cigarette was also the most commonly first-tried product in the 18–21 age group (Table 3; Fig. 3), with a mean age of 15.47. Most individuals in this age range, represented by Group 1 (*n* = 252.3), have tried every tobacco/nicotine product at least once. Participants in the second largest group, Group 3 (*n* = 115.5), reported cannabis as the first substance tried at a younger age (12.15), but again, only a very small number of participants showed this exceptional behavior. In Groups 1 and 4 (*n* = 28.2), hookah was the second product tried (15.71 and 16.21), following cigarette use (15.20 and 15.56).

**Table 3:** Demographic and substance use data (mean and %) by group for the 18-21-year-olds

		1	2	3	4	Abstinent	pvalue		
n		252.3336	19.9474	115.4671	28.2519	104.0000			
Age (mean)		19.5	19.5	19.8	19.9	19.4	0.563		
Gender	Cisgender female	51.5	60.3	44.9	67.9	52.6	< 0.05		
	Cisgender male	43.8	37.1	50.7	32.1	42.2			
	Transgender	4.7	2.5	4.4	0	5.1			
Perceived socioeconomic status	Above average	19.5	14.9	7.8	17.8	8.3	< 0.05		
	Average	61.1	74.1	76.8	73.1	74.3			
	Below average	19.5	10.9	15.4	9	17.4			
Lifetime consumption	Cigarette	95.8	57.7	67.2	82.2	N/A			
	E-cigarette	99.4	0	100	0	N/A			
	Hookah	76.8	91.8	57.1	6.6	N/A			
	Snus	54.7	0	0	21.9	N/A			
	Cannabis	88.5	39.6	2.1	58.7	N/A			
30-days consumption	Cigarette	56.8	24.7	25.7	29.9	N/A			
	E-cigarette	60.7	12.3	63.5	5.7	N/A			
	Hookah	12.2	11.1	16.5	0	N/A			
	Snus	11	0	0	0	N/A			
	Cannabis	30.8	30.7	1.4	13	N/A			
	Ecig-cig dual	40.2	12.3	17.1	5.7	N/A			

*The groups refer to the groups from* Fig. 3

*Group 1 has tried all nicotine / tobacco products with cigarette as the first product*

*Group 2 has tried cigarette*,* e-cigarette and cannabis with cigarette and cannabis as the first products*

*Group 3 has tried all nicotine / tobacco products except snus with cannabis as the first product*

*Group 4 has tried all nicotine / tobacco products except e-cigarette with cigarette as the first prodict*

**Fig. 4:**
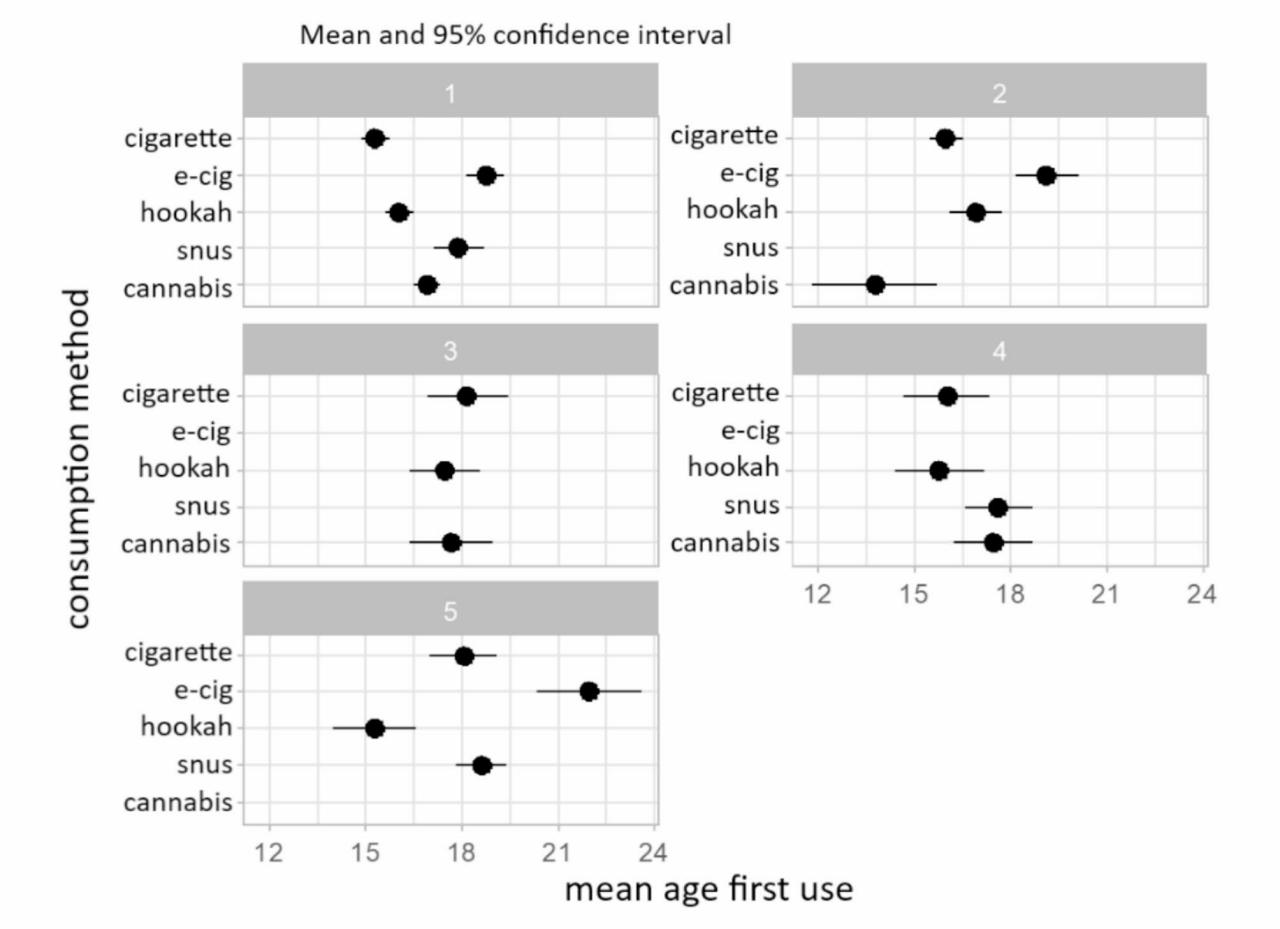
Mean age of each product used for the 22-25-year-olds divided into five groups

In the 22–25 age range (Table 4; Fig. 4), Group 1 (*n* = 231.2) was the largest group and reported cigarette as the first product tried (15.31), closely followed by hookah (16.07). Group 2 (*n* = 90.9) had cannabis as the first substance at a younger age (13.77). While this trend is exceptional, it is still present in the three groups. Hookah was the first- product for Group 3 (*n* = 63.8), Group 4 (*n* = 18.1), and Group 5 (*n* = 15.9), closely followed by cigarette. The mean age of cigarette use for 22-to-25-year-olds was 15.31.

**Table 4:** Demographic and substance use data (mean and %) by group for the 22-25-year-olds

		1	2	3	4	5	Abstinent	pvalue
n		231.2110	90.9278	63.8018	18.1195	15.9400	53.0000	
Age (mean)		23.5	23.6	23.6	23.3	23.5	23.3	0.502
Gender	Cisgender female	51.7	60.4	53.9	44.8	50.1	49.5	0.860
	Cisgender male	43.4	33.2	46.1	49.7	49.9	47.9	
	Transgender	4.9	6.3	0	5.4	0	2.6	
Perceived socioeconomic status	Above average	26.9	12.6	22.4	12.3	31.9	26.0	< 0.01
	Average	62	76.2	57.6	56.7	68.1	42.2	
	Below average	11.1	11.2	19.9	31	0	31.8	
Lifetime consumption	Cigarette	96.3	84.5	74.3	96.5	70.4	N/A	
	E-cigarette	100	100	0	0	34.2	N/A	
	Hookah	90.2	59.9	72	85.3	15.7	N/A	
	Snus	52.8	0	0	100	100	N/A	
	Cannabis	93.2	2.2	42.6	100	0	N/A	
30-days consumption	Cigarette	56.3	37.1	12.8	13	15.4	N/A	
	E-cigarette	56.3	52.7	0	0	26.8	N/A	
	Hookah	5.6	18.5	5.3	2.1	3.1	N/A	
	Snus	7.1	0	0	13.6	12.9	N/A	
	Cannabis	28.9	1	9.9	24.3	0	N/A	
	Ecig-cig dual	36.4	23.3	0	0	15.4	N/A	

*The groups refer to the groups from* Fig. 4

*Group 1 has tried all nicotine / tobacco products with cigarette as the first product.*

*Group 2 has tried all nicotine / tobacco products except snus with cannabis as the first product.*

*Group 3 has tried cigarette*,* hookah and cannabis with hookah as the first product.*

*Group 4 has tried all nicotine / tobacco products except e-cigarette with hookah as the first product.*

*Group 5 has tried all nicotine / tobacco products except cannabis with hookah as the first product.*

### Dual use of cigarette and e-cigarette

In the 14–17 age range (Table 2), the groups with the highest number of participants engaging in dual use of cigarette and e-cigarette are those that have tried most, if not all, tobacco products at least once: Group 1 (*n dual use* = 42.1) and Group 3 (*n dual use* = 29.2). This trend continues in the 18–21 age group (Table 3) with Group 1 (*n dual use* = 101.4) that has about half of its participants reporting dual use. Lastly, among the 22-25-year-olds (Table 4) dual users are least common, with Group 1 again having the highest number of dual users (*n dual use* = 84.1), representing just over a third of the group, and this group has also tried all tobacco/nicotine products at least once.

### Socioeconomic factors

In the overall sample, Group 1 (*n* = 688.2) had tried every substance, with transgender individuals being more represented in this group (5.5%). Cisgender females were more present (57.5%) in Group 3 (*n* = 61.9), which primarily used only cigarettes and hookah, and less present (47.7%) in the abstinent group (*n* = 248.0). Cisgender males were more represented in the abstinent group (48.8%) and Group 2 (45.4%), which had used cigarette, e-cigarette, and hookah. These gender differences were not significatively different across the age groups and no gender differences were found in the 22–25 age range. Regarding socioeconomic status, those reporting below-average status were more present in the non-consumer groups for the overall sample and the 22–25 age range. No differences based on the socioeconomic status was found for the 14–17 age group.

## Discussion

Overall, the mean age of initiation for nicotine/tobacco products was around 16 years old, which aligns with findings in the literature [11, 23]. The mean age of first consumption in the 14-17-age group was earlier than that of the among 18–25 age group, suggesting that consumption begins earlier in the younger generation. However, we cannot exclude the possibility of recall bias in the older groups. A study using data from 144 countries showed that 80% of adolescents started smoking before the age of 13 and that countries with lower-middle, and upper-middle income had a later age of cigarette initiation compared to low income countries [33], which is in line with our results.

Furthermore, in accordance with previous studies [34], conventional cigarettes were the primary initiation product, especially among 14-17-year-olds. This could be explained by the fact that they are the oldest products on the list and the most widely consumed in the general population. Additionally, conventional cigarettes may be more accessible to youth, whether obtained from friends (one cigarette in a package), through requests for others to purchase them, or even by theft from parents or other adults. Moreover, age verification during purchase is not consistently enforced across various points of sale [35].

However, e-cigarettes had a higher proportion of recent use compared to cigarettes, especially among the 14–17 age group, aligning with findings from prior studies [21, 36]. E-cigarette initiation tended to closely follow cigarette, a pattern already found in the literature although previous studies suggested that e-cigarette followed a couple of years later [37]. In our sample, e-cigarette followed cigarette very closely, a few months to a year. This can be attributed to the increasing prevalence and accessibility of e-cigarettes, which have emerged at earlier ages in more recent studies, including ours. Also, e-cigarettes were comparatively more affordable [38], partly because they were not included in the tobacco taxation law until 2024. While both e-cigarette and cigarette use were prevalent, we observed dual use among a minority of participants. However, the tendency for dual use appeared to decline with age, as very few respondents in the 22–25 age range reported being dual users.

Hookah was the primary medium for tobacco/nicotine initiation in the 22–25 age group. The preference for hookah as the first product tried among individuals in this generation may be attributed to a trend similar to the current popularity of e-cigarettes, which echoes the decrease of use observed in the HBSC study [8]. This attraction to hookah could, in part, be due to its variety of flavors, the appeal of smoke, and its social aspects. However, only one group reported a high proportion of e-cigarette use in the past month, while the other two groups shifted their focus to cannabis. Across all age groups, some individuals were identified as having cannabis as the first product with an early age of initiation, between 12 and 13 years old. This onset age is notably earlier compared to the mean age reported in the literature, which is typically around 16 years [15, 17]. This early initiation is rare but highlights the diversity of individual trajectories in substance use.

In the present study, the groups with the highest proportion of recent cannabis use in each age range were the participants with early age initiation. However, these groups were exceptions as most participants reported initiating cannabis between 16 and 17 years old, which aligns with previous findings in the literature [15, 17]. Notably, a group within the 18–21 age range also reported more recent consumption of cannabis compared to other products, which could be explained by early adulthood, a period during which cannabis consumption can increase, as indicated by prior research on dual use of cigarette and cannabis [39]. To understand why cannabis ranks as the third most used substance in this sample, it is important to note that early cannabis use has been associated with mental health symptoms [40]. It is possible be that cannabis is used as a coping mechanism for mental health issues. A Swiss study involving young men who underwent mandatory army conscription showed that those with attention deficit/hyperactivity disorder were more at risk of initiating cannabis and other substance use [41].

### Strengths and limitations

To our knowledge, this is the first study examining the initiation age of tobacco and/or nicotine products, including e-cigarettes and cannabis, in Switzerland. This research may pave the way for further studies on the topic and contribute to discussions on preventive measures regarding tobacco and/or nicotine use among the younger population.

However, the study has some limitations. Firstly, the questionnaire asked participants about their lifetime consumption before inquiring about their consumption in the past 30 days. The online format allowed respondents to navigate backward and change the answer to the lifetime question, potentially leading to inaccuracies in some proportions. Secondly, as previously stated, the question on conventional cigarettes also included cigars, cigarillo, and rolling tobacco use. However, only 3% of 15-24-year-olds reported one of the last three products mentioned as their initiation product [6]. Therefore, we can argue that the influence of cigarillos, cigars, and rolling tobacco had little impact on our sample.

Collecting data through social media also has its downside as people who do not fit the inclusion criteria may still respond to the survey. In addition, given that the initial focus was on disposable electronic nicotine delivery systems (also called *puffs*), users of these products might have been more inclined to participate in the questionnaire. The recruitment process via social media could also introduce bias, as it primarily targets young individuals with accounts; however a Swiss study has shown that approximately 98% of youth are active on at least one social media platform [42]. Furthermore, we were unable to use all social media platforms in our institution, including some of the most popular platforms among young people (Snapchat, TikTok). Therefore, even though the weightings helped to adjust the sample for some variables, it remains not fully representative of the 14–25 age population in French-speaking Switzerland. Lastly, the categorization of different groups in this study was determined by the authors, which has resulted in significant discrepancies in the number of respondents across certain groups.

## Conclusions

This study revealed that the mean age of initiation for all tobacco/nicotine products in French-speaking Switzerland in approximately 16 years old, with cigarette remaining the first one. Prevention campaigns should not overlook the ongoing presence of cigarettes in the initiation sequences of young smokers. However, e-cigarettes are a close second in usage across all age groups, often used alongside cigarettes, although the prevalence of dual use appears to decline with age. It is important to monitor both practices and the market, as adolescence is a vulnerable period for developing addictive behaviors, and the expanding availability of tobacco and/or nicotine products expands exposure risk. This highlights the necessity for policies to promote health and enhance awareness and knowledge about the risks of these products at a young age. To further protect the population, especially young people, effective tobacco control interventions such as raising tobacco taxes, banning tobacco advertising [43], implementing mass media campaigns, introducing plain packaging and enforcing stricter age verification measures should be implemented in Switzerland [1, 13]. While the minimum age for purchasing tobacco products and e-cigarettes was set at 18 throughout Switzerland in 2024, greater efforts are required to ensure compliance through more rigorous enforcement. Additionally, further studies are still needed to enable long-term monitoring and follow-up to capture substance initiation and use trends.

## Electronic supplementary material

Below is the link to the electronic supplementary material.

Supplementary Material 1

## Data Availability

The data that support the findings of this study are available from the corresponding author, upon reasonable request.

## Footnotes

1 The comma-separated numbers are explained by the application of weights.
