# Supplementary material for: Age of tobacco, nicotine and cannabis use initiation in Switzerland: a sequence analysis among adolescents and young adults
Source: BMC Public Health. 2024 Nov 19;24:3213. doi: 10.1186/s12889-024-20731-2 (PMC11575026; doi:10.1186/s12889-024-20731-2)
Supplement: Supplementary file 1 — Supplementary Material 1 [file 12889_2024_20731_MOESM1_ESM.docx]

You are aged…

- Under 14 years old
- 14 years old
- 15 years old
- 16 years old
- 17 years old
- 18 years old
- 19 years old
- 20 years old
- 21 years old
- 22 years old
- 23 years old
- 24 years old
- 25 years old
- Over 25 years old

What is your sex assigned at birth (biological sex) ?

- Female
- Male
- Intersex

What is your gender identity (what you feel inside, how you define yourself)?

- Female
- Male
- Other, please specify

Were you born in Switzerland?

- Yes
- No

In which country were you born? Please write your answer here:

You live in the canton of...

- Bern
- Fribourg
- Geneva
- Jura
- Neuchâtel
- Valais
- Vaud
- Other, please specify:

Your place of residence (where you spend most of your time) is…

- In the mountains, countryside, village (less than 10,000 inhabitants)
- In a city, suburban area (more than 10,000 inhabitants)

What is your current living arrangement (where you spend most of your time)?

- With my mother and father
- With my mother and stepfather
- With my father and stepmother
- Only with my mother
- Only with my father
- With a family member (grandparents, uncle, aunt, etc.)
- With my partner (married or not)
- Alone
- In a shared apartment
- In a student residence
- In a social institution (home, orphanage, etc.)
- Other, please specify

What is your parents' current situation?

- They live together
- They are separated or divorced
- Your father is deceased
- Your mother is deceased
- Both your father and mother are deceased
- Other, please specify

How much money (in Swiss Francs) do you personally have per month on average (including pocket money, salary, etc.)? Please write your answer here:

Compared to other families in Switzerland, do you think your family’s financial situation is…

- Above average
- Average
- Below average

What is your main activity?

- Compulsory school
- Post-compulsory school (high school, commercial school, etc.)
- Special education school
- Higher education (HE)
- University / College
- Pre-apprenticeship / transition school / pre-professional school
- Apprenticeship (CFC)
- Work / employment
- No activity (e.g., gap year)
- Looking for work
- Other, please specify

What have you already used at least once in your life...?

- Cigarette / rolling tobacco / cigar / cigarillos
- Electronic cigarette (e.g., vape, puff, e-shisha)
- Shisha / water pipe / hookah
- Snus, snuff or chewing tobacco
- Tobacco-free nicotine pouch
- Heated tobacco (e.g., IQOS)
- Cannabis (e.g., joint, hash, marijuana, hashish)
- None of the above substances

At what age did you first use a cigarette / rolling tobacco / cigar / cigarillos? (If you don't remember, please indicate: 0) Please write your answer here:

At what age did you first use an electronic cigarette (e.g., vape, puff, e-shisha)? (If you don't remember, please indicate: 0) Please write your answer here:

At what age did you first use a shisha / water pipe / hookah? (If you don't remember, please indicate: 0) Please write your answer here:

At what age did you first use snus, snuff or chewing tobacco? (If you don't remember, please indicate: 0) Please write your answer here:

At what age did you first use a tobacco-free nicotine pouch? (If you don't remember, please indicate: 0) Please write your answer here:

At what age did you first use heated tobacco (e.g., IQOS)? (If you don't remember, please indicate: 0) Please write your answer here:

At what age did you first use cannabis (e.g., joint, hash, marijuana, hashish)? Please write your answer here:

What have you used in the last 30 days...?

- Cigarette / rolling tobacco / cigar / cigarillos
- Electronic cigarette (e.g., vape, puff, e-shisha)
- Shisha / water pipe / hookah
- Snus, snuff or chewing tobacco
- Tobacco-free nicotine pouch
- Heated tobacco (e.g., IQOS)
- Cannabis (e.g., joint, hash, marijuana, hashish)
- None of the above substances

…

*A part of the questionnaire is not shown as it was not used for this study.*

…

Frankly, do you think your answers are sincere enough to be used?

- Yes
- No
